# Use of Tumor-infiltrating lymphocytes (TILs) to predict the treatment response to eribulin chemotherapy in breast cancer

**DOI:** 10.1371/journal.pone.0170634

**Published:** 2017-02-06

**Authors:** Shinichiro Kashiwagi, Yuka Asano, Wataru Goto, Koji Takada, Katsuyuki Takahashi, Satoru Noda, Tsutomu Takashima, Naoyoshi Onoda, Shuhei Tomita, Masahiko Ohsawa, Kosei Hirakawa, Masaichi Ohira

**Affiliations:** 1 Department of Surgical Oncology; Osaka City University Graduate School of Medicine, Osaka, Japan; 2 Department of Pharmacology, Osaka City University Graduate School of Medicine, Osaka, Japan; 3 Department of Diagnostic Pathology; Osaka City University Graduate School of Medicine, Osaka, Japan; University of South Alabama Mitchell Cancer Institute, UNITED STATES

## Abstract

**Background:**

Eribulin mesylate (eribulin) is currently indicated for treatment of locally advanced or metastatic breast cancer (MBC). It is a cytotoxic agent with unique mechanisms that suppress epithelial-mesenchymal transition (EMT) of cancer cells. On the other hand, Tumor-infiltrating lymphocytes (TILs), which are considered indicators of immune response monitoring, have been reported as prognostic factors and predictors of therapeutic efficacy. We thought that eribulin, which has an EMT-inhibiting mechanism, may produce an antitumor effect by improving the immune microenvironment, and in this study investigated the effects of breast cancer eribulin chemotherapy on the immune microenvironment with TILs as a marker.

**Methods:**

TILs was evaluated in 52 patients with MBC who underwent chemotherapy with eribulin. The correlation between TILs evaluated according to the standard method, and prognosis, including the efficacy of eribulin chemotherapy, was investigated retrospectively.

**Results:**

Of the 52 MBC patients, 29 (55.8%) were in the high TILs group and 23 (44.2%) were in the low TILs group. The high TILs group included significantly more triple-negative breast cancer (TNBC) (p = 0.008) than the low TILs group. In an analysis of outcomes, TNBC patients in the high TILs group had significantly longer disease-free survival than TNBC patients in the low TILs group (p = 0.033, log-rank), but no significant differences were seen in all breast cancer patients (p = 0.489, log-rank) or in non-TNBC patients (p = 0.878, log-rank). In a multivariate analysis of recurrence in TNBC patients, being in the high TILs group was again an independent factor for a good outcome (p = 0.031, HR = 0.063).

**Conclusion:**

The results of this study suggest that TILs may be useful as a predictive marker of the therapeutic effect of eribulin chemotherapy in TNBC.

## Introduction

Eribulin mesylate (eribulin) stops cell division by inhibiting microtubule extension [1–3], and has a mechanism of action that differs from other antimitotic drugs such as taxane and vinca alkaloids [2, 4, 5]. Thus, eribulin binds to microtubule ends and suppresses microtubule polymerization. Taxane binds extensively inside microtubules and suppresses shortening of microtubules by depolymerization. Vinca alkaloids bind to the external surface of microtubules and suppress both microtubule polymerization and depolymerization. Consequently, the anti-cancer effect differs among these agents. For example, in a phase III trial of eribulin (EMBRACE, Eisai Metastatic Breast Cancer Study Assessing Physician’s Choice versus E7389), a significant prolongation of overall survival was observed in patients with locally advanced or metastatic breast cancer (MBC) after eribulin treatment even without an improvement in disease free survival [6]. This effect was partially explained by a decrease in the occurrence of new metastatic lesions with eribulin therapy, an effect that has not been demonstrated with other drugs. However, the precise mechanism of this clinically significant benefit has not yet been elucidated. Some of the unique anticancer effects of eribulin have emerged from experimental studies using cancer cells and tumor tissues [7, 8]. These include suppression of the epithelial-mesenchymal transition (EMT) of cancer cells and promotion of vascular remodeling in tumors.

Stephen Paget proposed the “seed and soil” theory with regard to cancer metastases in 1889, and, since that time, the importance of the tumor microenvironment for cancer cell proliferation has been increasingly recognized [9, 10]. Tumor tissue is composed not only of cancer cells, but also inflammatory cells, immunocytes, vascular and lymphatic cells, fibroblasts, and fibrous tissue, and these elements comprise the characteristic tumor microenvironment. The importance of regulating and improving the immune microenvironment in cancer has been recognized because the immune microenvironment in cancer tissues affects not only the efficacy of immunotherapy, but also the efficacy and prognosis of conventional chemotherapy and other modes of anticancer therapy [11, 12]. Therefore, monitoring the host's immune response to cancer in the microenvironment is believed to play a key role in predicting therapeutic efficacy and prognosis. Tumor-infiltrating lymphocytes (TILs), which are considered indicators of immune response monitoring, have been reported as prognostic factors and predictors of therapeutic efficacy [13–15].

The progression of cancer is not determined solely by the properties of the cancer cells themselves; it is also closely associated with the interrelation between cancer cells and their microenvironment, including EMT and immune responses. EMT suppression seems to contribute to improving the immune microenvironment [16]. We therefore thought that eribulin, which has an EMT-inhibiting mechanism, may produce an antitumor effect by improving the immune microenvironment, and in this study investigated the effects of breast cancer eribulin chemotherapy on the immune microenvironment with TILs as a marker.

## Materials and methods

### Patient background

The subjects included 52 patients with MBC who underwent chemotherapy using eribulin from August 2011 to June 2013 at our institute. The median follow-up time was 431 days (range, 50–650 days). The overall response rate (ORR), clinical benefit rate (CBR), disease control rate (DCR), overall survival (OS), time to treatment failure (TTF) and progression-free survival (PFS) were calculated regarding the efficacy of this regimen. The TTF was evaluated on a daily basis and set as the period from the date of treatment commencement to cancellation for any reason, including disease aggravation, treatment toxicity and death. The OS was evaluated on a daily basis and set as the period from the date of treatment commencement to death. The PFS was evaluated on a daily basis and set as the period from the date of treatment commencement to either the earlier of the date of death or confirmation of progressive disease (PD).

Regarding the outline of the chemotherapy regimen, one course of treatment consisted of 21 days (three weeks). Eribulin mesylate (1.4 mg/m^2^) was intravenously administered on days 1 and 8, after which a withdrawal period was continued to day 21 [6]. This protocol was repeated until PD was detected or a severe adverse event requiring the discontinuation of the scheduled chemotherapy was noted. The chemotherapy was administered on an outpatient basis in all cases. The antitumor effect was evaluated based on the criteria for therapeutic effects conforming to the RECIST criteria (Response Evaluation Criteria in Solid Tumors) version 1.1 [17].

The morphology of the tumor, including the histological tissue type, nucleus grade, etc., was evaluated using conventional hematoxylin and eosin (HE) staining. Moreover, breast cancer was classified into subtypes according to the immunohistochemical expression of the estrogen receptor (ER), progesterone receptor (PgR), human epidermal growth factor receptor 2 (HER2) and Ki67. Based on their immunohistochemical expression, the tumours are categorized into the immunophenotypes luminal A (ER+ and/or PgR+, HER2-, Ki67-low), luminal B (ER+ and/or PgR+, HER2+) (ER+ and/or PgR+, HER2-, Ki67-high), HER2-enriched (ER-, PgR-, and HER2+), and triple-negative breast cancer (TNBC) (negative for ER, PgR and HER2) [18].

### Ethics statement

The design of this study is a retrospective chart review study. Written informed consent was obtained from all subjects. This research conformed to the provisions of the Declaration of Helsinki in 2013. All patients were informed of the investigational nature of this study and provided their written, informed consent. The study protocol was approved by the Ethics Committee of Osaka City University (#926).

### Histopathological evaluation

**Fig 1:**
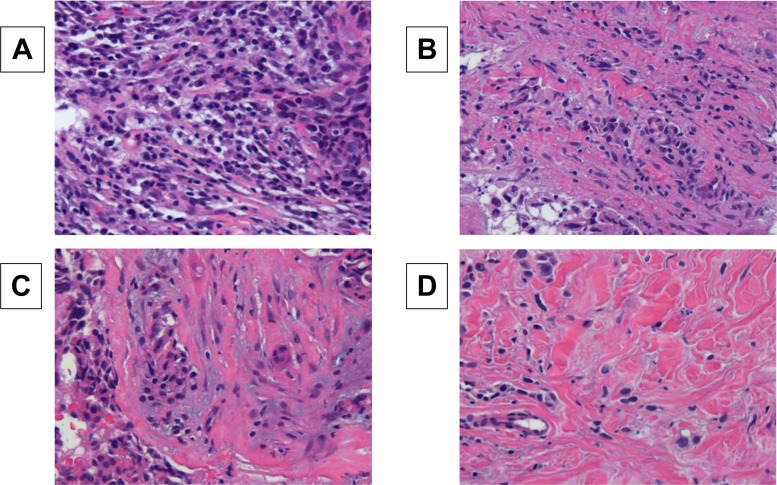
Histopathologic analysis of the percentage of TILs was performed on a single full-face hematoxylin and eosin-stained tumor section. TILs were defined as the percentage of tumor stroma containing infiltrating lymphocytes. Proportional scores were defined as 3, 2, 1, and 0 if the area of stroma with lymphoplasmacytic infiltration around invasive tumor cell nests was > 50% (A); > 10–50% (B); ≤ 10% (C); and absent (D), respectively.

Histopathological assessment of predictive factors was made for core needle biopsy (CNB) specimens for primary lesions at the time of the breast cancer diagnosis. Histopathologic analysis of the percentage of TILs was evaluated on a single full-face hematoxylin and eosin (HE)-stained tumor section using criteria described by Salgado et al [19]. TILs were defined as the infiltrating lymphocytes within tumor stroma and were expressed in proportion to the field investigated [19–21]. The area of in situ carcinoma and crush artifacts were not included. Proportional scores were defined as 3, 2, 1, and 0 if the area of stroma with lymphoplasmacytic infiltration around invasive tumor cell nests was > 50%, > 10–50%, ≤ 10%, and absent, respectively **(Fig 1)**. TILs were considered positive when scores were ≥ 2, and negative when scores were 1 and 0. Histopathologic evaluation of TILs was jointly performed by two breast pathologists, who were blinded to clinical information, including treatment allocation and outcomes.

### Statistical analysis

Statistical analysis was performed using the SPSS^®^ version 19.0 statistical software package (IBM, Armonk, NY, USA). Categorical data are reported with numbers and percentages, and continuous data as a median and range. The association between TILs and other clinicopathological variables, and the significance of different prognostic markers were analysed using the chi-squared test (or Fisher’s exact test when necessary). The association with survival was analysed using the Kaplan–Meier plot and log-rank test. The Cox proportional hazards model was used to compute univariate and multivariate hazard ratios (HR) for the study parameters with 95% confidence intervals (CI), and used in a backward stepwise method for variable selection in multivariate analysis. In all of the tests, a *p*-value of less than 0.05 was considered statistically significant. Cut-off values for different biomarkers included in this study were chosen before statistical analysis.

## Results

### Clinical effects of eribulin chemotherapy

The subjects included 52 patients who underwent chemotherapy using eribulin against MBC. The clinical effects were as follows: ORR = 34.6% (18/52); CBR = 44.2% (23/52); DCR = 51.9% (27/52); median OS = 334 days; median TTF = 81 days; and median PFS = 275 days. The distribution of the intrinsic subtype classification was as follows: Luminal A = 12 cases (23.1%); Luminal B = 13 cases (15.0%); Luminal HER2 = 2 cases (3.8%); HER2 enriched = 3 cases (5.8%) (non-TNBC 30 cases, 57.7%); and TNBC = 22 cases (42.3%). In an investigation according to the intrinsic subtype, the respective ORR was found to be 40.0% (12/30) in the non-TNBC cases and 27.3% (6/22) in the TNBC cases **(S1 Table)**.

### Tumor-infiltrating lymphocytes in eribulin chemotherapy cases

TILs were determined in every sample and ranged from 0 to 88 (mean, 15; median, 18; standard deviation 5). Of the 52 patients, 29 (55.8%) were in the high TILs group and 23 (44.2%) were in the low TILs group. The high TILs group included significantly more TNBC (p = 0.008) than the low TILs group, but no correlations were seen with any other clinicopathological factors **(Table 1)**. TILs were not correlated with any clinicopathological factors in either TNBC or non-TNBC.

**Table 1:** Correlations between tumor-infiltrating lymphocytes and clinicopathological parameters in 52 locally advanced or metastatic breast cancers and their Triple negative- and non-Triple negative-subtypes.

Parameters	All breast cancer (n = 52)			Triple-negative (n = 22)			non-Triple-negative (n = 30)		
	High (n = 29)	Low (n = 23)	p value	High (n = 17)	Low (n = 5)	p value	High (n = 12)	Low (n = 18)	p value
Estrogen receptor									
Negative	16 (55.2%)	7 (30.4%)							
Positive	13 (44.8%)	16 (69.6%)	0.074						
Progesterone receptor									
Negative	20 (69.0%)	12 (52.2%)							
Positive	9 (31.0%)	11 (47.8%)	0.216						
HER2									
Negative	26 (89.7%)	21 (91.3%)							
Positive	3 (10.3%)	2 (8.7%)	0.612						
HR and HER2 status									
TNBC	17 (58.6%)	5 (21.7%)							
non-TNBC	12 (41.4%)	18 (78.3%)	0.008						
Age at chemotherapy									
≤63	13 (44.8%)	13 (56.5%)		9 (52.9%)	3 (60.0%)		4 (33.3%)	10 (55.6%)	
>63	16 (55.2%)	10 (43.5%)	0.402	8 (47.1%)	2 (40.0%)	0.594	8 (66.7%)	8 (44.4%)	0.206
Degree of progress									
Locally advanced	8 (27.6%)	5 (21.7%)		5 (29.4%)	1 (20.0%)		3 (25.0%)	4 (22.2%)	
Visceral metastases	21 (72.4%)	18 (78.3%)	0.629	12 (70.6%)	4 (80.0%)	0.581	9 (75.0%)	14 (77.8%)	0.597
Life threatening condition									
non- Life threatening	21 (72.4%)	17 (73.9%)		11 (64.7%)	2 (40.0%)		10 (83.3%)	15 (83.3%)	
Life threatening	8 (27.6%)	6 (26.1%)	0.904	6 (35.3%)	3 (60.0%)	0.316	2 (16.7%)	3 (16.7%)	0.696
Nuclear grade									
1, 2	16 (55.2%)	17 (73.9%)		4 (23.5%)	1 (20.0%)		12 (100.0%)	16 (88.9%)	
3	13 (44.8%)	6 (26.1%)	0.163	13 (76.5%)	4 (80.0%)	0.687	0 (0.0%)	2 (11.1%)	0.352
Ki67									
Negative	13 (44.8%)	13 (56.5%)		7 (41.2%)	4 (80.0%)		6 (50.0%)	9 (50.0%)	
Positive	16 (55.2%)	10 (43.5%)	0.402	10 (58.8%)	1 (20.0%)	0.155	6 (50.0%)	9 (50.0%)	0.645

HR, hormone receptor. HER2, human epidermal growth factor receptor 2. TNBC, triple-negative breast cancer.

**Fig 2:**
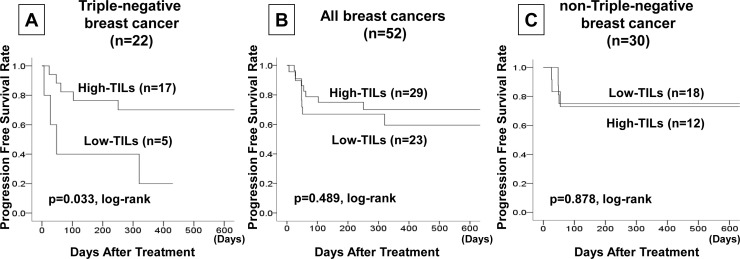
In an analysis of outcomes, TNBC patients in the high TILs group had significantly longer disease-free survival than TNBC patients in the low TILs group (p = 0.033, log-rank) **(A)**, but no significant differences were seen in all breast cancer patients (p = 0.489, log-rank) **(B)** or in non-TNBC patients (p = 0.878, log-rank) **(C)**.

**Fig 3:**
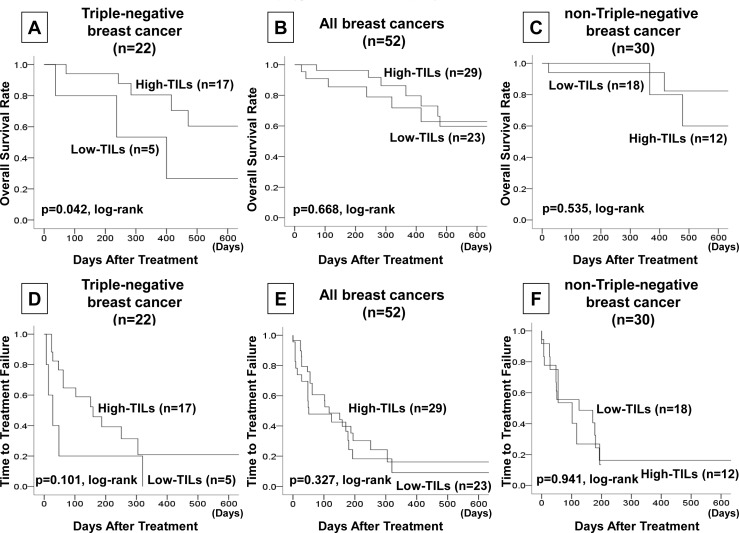
Among TNBC patients OS was significantly longer in the high TILs group than in the low TILs group (p = 0.042, log-rank) **(A)**. However, no increase in OS was seen among all breast cancer patients (p = 0.668, log-rank) **(B)** or among non-TNBC patients (p = 0.535, log-rank) **(C)**. With regard to TTF, no significant differences were seen in any subtype **(D–F)**.

In an analysis of outcomes, TNBC patients in the high TILs group had significantly longer disease-free survival than TNBC patients in the low TILs group (p = 0.033, log-rank), but no significant differences were seen in all breast cancer patients (p = 0.489, log-rank) or in non-TNBC patients (p = 0.878, log-rank) **(Fig 2A–2C)**. Similarly, among TNBC patients OS was significantly longer in the high TILs group than in the low TILs group (p = 0.042, log-rank) **(Fig 3A–3C)**. However, no increase in OS was seen among all breast cancer patients (p = 0.668, log-rank) or among non-TNBC patients (p = 0.535, log-rank). With regard to TTF, no significant differences were seen in any subtype **(Fig 3D–3F)**.

In a univariate analysis of recurrence in TNBC patients, being in the high TILs group was a factor for a good outcome (p = 0.047, HR = 0.260). In a multivariate analysis, being in the high TILs group was again an independent factor for a good outcome (p = 0.031, HR = 0.063) **(Table 2)**.

**Table 2:** Univariate and multivariate analysis with respect to progression free survival in 22 triple-negative breast cancers.

		Univariate analysis			Multivariate analysis		
Parameters		Hazard ratio	95% CI	p value	Hazard ratio	95% CI	p value
Age at chemotherapy	≤63vs >63	0.470	0.117–1.893	0.288			
Degree of progress	Locally advanced vs Visceral metastases	1.109	0.230–5.352	0.898			
Life threatening condition	non- Life threatening vs Life threatening	1.720	0.460–6.427	0.420			
Nuclear grade	1, 2, vs 3	2.915	0.364–23.352	0.314	2.045	0.208–20.120	0.540
Ki67	≤14% vs >14%	1.368	0.364–5.133	0.642	5.736	0.438–75.058	0.183
TILs	High vs Low	0.260	0.069–0.980	0.047	0.063	0.005–0.771	0.031

CI, confidence interval. TILs, tumor-infiltrating lymphocytes.

## Discussion

EMT is observed when cancer spreads, and promotes cancer infiltration and metastasis by facilitating the ability of cancer cells to move and the breakdown of the extracellular matrix [22]. Cancer cells with induced EMT are known to acquire treatment resistance and to have enhanced properties as cancer stem cells [23]. It is also reported that inhibiting EMT improves the cancer immune microenvironment and enhances the antitumor immune response [24]. An enhanced antitumor immune response contributes not only to immunotherapy but also to the antitumor effect of conventional chemotherapy [11]. Thus, inhibition of EMT with eribulin chemotherapy is thought to enhance the antitumor immune response via improvement in the cancer immune microenvironment.

Among the intrinsic subtypes of breast cancer, eribulin chemotherapy is also reported to be particularly useful for TNBC [25, 26]. In recent years it has been shown that TNBC can be subdivided into 7 different subtypes according to gene expression profile [27–29]. Among them are mesenchymal (M) and mesenchymal-stem like (MSL) subtypes that have high levels of expression of EMT-related genes (also high expression levels of stem cell-related genes). Eribulin plays a role in EMT inhibition, and seems promising as a drug that is effective against these subtypes of TNBC.

In this study, TILs were significantly higher in TNBC patients than in non-TNBC patients. High levels of TILs, a marker for monitoring the antitumor immune response, suggest a high level of immune activity in TNBC patients. In the TNBC subtype classification, there is an immunomodulatory (IM) subtype with high expression levels of genes related to immune response [27], and it may be that cases with high levels of TILs are related to these subtypes.

In an analysis of outcomes among TNBC patients, longer PFS and OS were seen in the high TILs group than in the low TILs group. The Kaplan-Meier curve in this investigation showed a characteristic delayed separation curve in immunotherapy in the high TILs group, and despite the short follow-up time, one may conjecture that eribulin chemotherapy contributes to the antitumor immune response. The enhanced antitumor immune response that accompanies EMT suppression with eribulin chemotherapy may be behind the longer OS in the EMBRACE trial [6, 26]

In breast cancer chemotherapy, TILs are thought to be useful as a marker to predict the therapeutic effect in TNBC and HER2-positive breast cancers [13–15, 30]. However, these reports are with anthracycline, taxanes, platinum-based agents, and trastuzumab; the clinical relevancy of eribulin and TILs has yet to be demonstrated. This study had a small number of patients, and, although the HER2-positive breast cancer data could not be confirmed, the study showed that in TNBC the antitumor immune response could be monitored with TILs. The ability to predict the therapeutic effect of eribulin chemotherapy with TILs would seem to be promising in that it could select only those patients who would respond to combination therapy with eribulin chemotherapy and immune therapy.

## Conclusions

The results of this study suggest that TILs may be useful as a predictive marker of the therapeutic effect of eribulin chemotherapy in TNBC.

## Supporting information

S1 Table Clinical effects of eribulin chemotherapy in breast cancer subtypes. The clinical effects were as follows: overall ORR = 34.6% (18/52); CBR = 44.2% (23/52); DCR = 51.9% (27/52). In an investigation according to the intrinsic subtype, the respective ORR was found to be 40.0% (12/30) in the non-TNBC cases and 27.3% (6/22) in the TNBC cases.
(DOCX)
