# Supplementary material for: Use of Tumor-infiltrating lymphocytes (TILs) to predict the treatment response to eribulin chemotherapy in breast cancer
Source: PLoS One. 2017 Feb 6;12(2):e0170634. doi: 10.1371/journal.pone.0170634 (PMC5293550; doi:10.1371/journal.pone.0170634)
Supplement: S1 Table — The clinical effects were as follows: overall ORR = 34.6% (18/52); CBR = 44.2% (23/52); DCR = 51.9% (27/52). In an investigation according to the intrinsic subtype, the respective ORR was found to be 40.0% (12/30) in the non-TNBC cases and 27.3% (6/22) in the TNBC cases. (DOCX) [file pone.0170634.s001.docx]

**S1 Table. Clinical effects of eribulin chemotherapy in breast cancer subtypes.**

|  | All breast cancer　(n=52) | Triple-negative (n=22, 42.3 %) | non-Triple-negative (n=30, 57.7 %) |
| --- | --- | --- | --- |
| CR; Complete Response | 1 (1.9 %) | 0 (0.0 %) | 1 (3.3 %) |
| PR; Partial Response | 17 (32.7 %) | 6 (27.3 %) | 11 (36.7 %) |
| Long SD; Stable Disease >24weeks | 5 (9.6 %) | 2 (9.1 %) | 3 (10.0 %) |
| SD; Stable Disease | 4 (7.7 %) | 1 (4.5%) | 3 (10.0 %) |
| PD; Progressive Disease | 20 (38.5 %) | 13 (59.1 %) | 7 (23.3 %) |
| NE; Not Evaluable | 5 (9.6 %) | 0 (0.0 %) | 5 (16.7 %) |
| ORR; Objective Response Rate (CR + PR) | 18 (34.6 %) | 6 (27.3 %) | 12 (40.0 %) |
| CBR; Clinical Benefit Response (CR + PR + Long SD) | 23 (44.2 %) | 8 (36.4 %) | 15 (50.0 %) |
| DCR; Disease Control Rate (CR + PR + SD) | 27 (51.9 %) | 9 (40.9 %) | 18 (60.0 %) |
